# Development and Validation of a New TaqMan Real-Time PCR for the Detection of *Ornithobacterium rhinotracheale*

**DOI:** 10.3390/microorganisms10020341

**Published:** 2022-02-01

**Authors:** Amro Hashish, Avanti Sinha, Yuko Sato, Nubia R. Macedo, Mohamed El-Gazzar

**Affiliations:** 1 Department of Veterinary Diagnostic and Production Animal Medicine, College of Veterinary Medicine, Iowa State University, Ames, IA 50011, USA; hashish@iastate.edu (A.H.); asinhac@gmail.com (A.S.); ysato@iastate.edu (Y.S.); nubia@iastate.edu (N.R.M.); 2 Agriculture Research Center, National Laboratory for Veterinary Quality Control on Poultry Production, Animal Health Research Institute, Giza 12618, Egypt

**Keywords:** *Ornithobacterium rhinotracheale* (ORT), ornithobacteriosis, TaqMan real-time PCR (qPCR), bacterial detection, clinical samples

## Abstract

*Ornithobacterium rhinotracheale* (ORT) has been associated with poultry respiratory disease worldwide. The organism is fastidious and isolation is challenging. One TaqMan real-time PCR (qPCR) assay has been developed for the detection of ORT. However, during validating the ORT qPCR, the assay performance was suboptimal. During the in silico evaluation, deviations from the basic parameters for primers and probes designs (e.g., presence of stable undesirable primer-dimers) were observed. The suboptimal design led to low efficiency and low sensitivity of the assay. Initially, modification on the probe was carried out to improve the performance of the assay. However, the assay’s performance (efficiency and sensitivity) was still suboptimal. In this manuscript, we describe the development of a new qPCR assay and the comparison of its performance with the currently available assay. A highly efficient, sensitive, and specific qPCR assay was developed with approximately 1000-folds reduction in the limit of detection (from 3 × 10^6^ plasmid DNA copies/mL to 1 × 10^3^ plasmid DNA copies/mL). Additionally, the efficiency of the new assay (E = 98.70%) was significantly better than the current assay (E = 73.18%). The newly developed assay is an improved diagnostic tool for the sensitive and efficient diagnosis of ORT from clinical samples.

## 1. Introduction

Bacterial respiratory infections are one of the most significant challenges facing the poultry industry in the USA and worldwide [1,2]. They lead to significant economic losses due to high condemnation rates, increased mortality, and production losses [2,3]. *Ornithobacterium rhinotracheale* (ORT) is one of the most important bacterial respiratory pathogens primarily facing the turkey industry. In 2020, ORT was ranked fourth in the list of the top health issues in the American turkey industry, and it has been consistently fluctuating between number 3 and number 4 for the past several years [4].

*Ornithobacterium rhinotracheale* is a Gram-negative, non-motile, non-sporulating bacterium [5]. Currently, 18 ORT serotypes (A–R) have been reported [6,7]. *O. rhinotracheale* affects turkeys, chickens, and other domestic and wild avian species [7]. The clinical picture of ornithobacteriosis in turkeys and chickens is not pathognomonic, and ORT presents itself similar to many other respiratory diseases caused by bacterial or viral pathogens [5,7]. Therefore, relying on clinical signs and postmortem lesions is of little diagnostic value, and confirmatory testing is required. Confirmatory diagnosis of ORT infection can be reached through isolation and/or molecular detection [5]. Isolation is useful; however, due to the fastidious nature of ORT and the lack of commercially available specific or selective media, it is often overgrown by commensals and/or other pathogens [5,8]. This increases the number of flocks where ORT diagnosis cannot be confirmed [8]. Molecular diagnosis of ORT directly from clinical samples offers a superior alternative. Moreover, it provides several advantages over bacterial culture, including higher sensitivity and specificity and less turnaround time [9]. Currently, two published PCR assays have been developed for the diagnosis of ORT [6,10]. The first assay is a conventional PCR targeting 784 bp of the16S ribosomal ribonucleic acid (rRNA) gene [6]. This conventional PCR assay was used widely to diagnose ORT [11,12,13,14]; however, false-positive results, particularly with *Pasteurella multocida*, were reported [6,11], and the sensitivity was reported to be unsatisfactory [6]. The second assay is a TaqMan real-time PCR (qPCR) that was designed to target the same gene [10].

Primers and probe are the primary determinants of any qPCR assay’s performance. Suboptimal design of primers and probe severely impairs the performance of any assay [15,16,17]. In silico analysis of the primers and probe of the currently available ORT TaqMan qPCR assay [10] revealed potential ways to improve the assay’s performance. The presence of guanine (G) base was observed at the 5′ end of the probe adjacent to the reporter dye, which negatively impacts the fluorogenic probe [18] as it quench the fluorophore [16,19]. Additionally, in silico analysis of the primers revealed the formation of primer-dimers with high Gibbs Free Energy (ΔG), the energy required to break the secondary structure [20], with negative values indicating stable undesirable secondary structures. These parameters can explain the reduced assay’s efficiency below the acceptable range, and consequently, the increased limit of detection as was tested against different 10-fold serial dilutions of ORT isolates and clinical samples. In the initial manuscript, the reported efficiency for this assay was 100% [10], which is in disagreement with the performance of the assay in our hands and in disagreement with the in silico analysis of the designed primers.

The performance and the described problems with the primers and probe design of this assay made it an unreliable diagnostic tool to be validated and to be further used for the detection of ORT. In the present study, we initially aimed to modify the probe of the current TaqMan qPCR [10] (this assay will be referred to “modified probe assay” throughout the manuscript) in a trial to improve its performance; however, the improvements in performance were insufficient. Therefore, a completely new assay targeting the same 16S rRNA gene segment was developed (this assay will be referred to “newly developed assay” throughout the manuscript). Additionally, a comparison between the performances of the three assays: the current assay, the modified probe assay, and the newly developed assay, was performed.

## 2. Materials and Methods

### 2.1. Primers and Probes Design and Modification

The probe of the currently available TaqMan qPCR assay was modified by removing the first 5′ end G base (Table 1). Primers of the new assay were designed to target 131 bp of the 16S rRNA gene (the same target gene of the currently available assay). The designed primers and probe were tested for their specificity through in silico analysis using the BLAST search tool [21]. To avoid the formation of secondary structure and primer-dimers, the online IDT oligo Analyzer 3.1 tool (https://www.idtdna.com/calc/analyzer) (accessed on 13 December 2020) was used to analyze the newly designed primers and probe.

All oligonucleotides included in this study (primers and probes) were synthesized by IDT (Integrated DNA Technologies, Coralville, IA, USA). The sequences and characteristics of the primers and probes included in this study are displayed in Table 1.

### 2.2. Real-Time PCR Setup

To conduct a fair comparison between the three qPCR assays, we intentionally used the same qPCR conditions and reagents to run all assays. Primers and probes were mixed in a 20 µL reaction containing 5 µL of TaqMan Fast Virus 1-step MM (Applied Biosystems, Carlsbad, CA, USA), primers to a final concentration of 0.4 µmol, probe to a final concentration of 0.2 µmol, 8.145 µL of water, and 5 µL of DNA template.

Each reaction was conducted in Real-Time PCR System 7500 (Applied Biosystems, Carlsbad, CA, USA). Based on the calculated T_m_ of the primers and probes, the following amplification conditions were adopted: 50 °C for 5 min; 95 °C for 20 s with optics off; 40 cycles of 95 °C for 15 s, followed by 60 °C for 60 s with optics on. These adopted amplification conditions were slightly different from the conditions described in the initial manuscript of the current assay (95 °C for 15 min, 42 cycles of 94 °C for 60 s, and 60 °C for 60 s [10]. However, the annealing-extension step was the same “60 °C for 60 s”.

A non-template control (PCR-grade H_2_O) and a positive control (isolated DNA from ORT isolate confirmed by matrix-assisted laser desorption ionization time-of-flight mass spectrometry “MALDI-TOF”) were included in each run. All results were analyzed using SDS 1.5.1 software (Applied Biosystems, Carlsbad, CA, USA).

### 2.3. Ornithobacterium rhinotracheale Isolates and Clinical Samples

Thirty-eight ORT isolates were included in this study (Table 2). These isolates were revived on blood agar (with 5% sheep blood) and incubated for 24–48 h under microaerophilic conditions at 37 °C for 48 h. Characteristic ORT colonies were confirmed by MALDI-TOF [22]. Additionally, nine known ORT-positive clinical samples were obtained from ISU-VDL (Table 3). Subsequent confirmation of these ORT clinical samples was performed through bacteriological isolation of ORT. Homogenates of tracheas and lungs from apparently normal chickens and turkeys (negative ORT flocks by isolation) were also included (Table 3).

### 2.4. Other Bacteria and Viruses

Eighteen respiratory microorganisms (twelve bacterial and six viral isolates) were included to test the assays’ analytical specificity. All microorganisms included in this study and their growth conditions are listed in Table 2.

### 2.5. Nucleic Acid Extraction

Each of the grown bacterial isolates was re-suspended in 1 mL phosphate-buffered saline. Clinical swabs were prepared by pooling 5 swabs in 1 mL phosphate-buffered saline. Clinical tissue homogenates were prepared using Geno/Grinder automated homogenizer following the instructions of the manufacturer. An amount of 100 µL of bacterial resuspension, viral media, swab resuspension, or tissue homogenate was used for the extraction of nucleic acid from each of the listed samples (Table 2 and Table 3). Nucleic acid extraction was conducted using a MagMAX™ Pathogen RNA/DNA Kit (Thermo Fisher Scientific, Waltham, MA, USA) on a Kingfisher-Flex instrument (Thermo Fisher Scientific, Waltham, MA, USA ) following the instructions of the manufacturer. Nucleic acids were eluted into 90 μL of elution buffer.

### 2.6. Evaluation of qPCR Assays’ Performance

#### 2.6.1. In Silico Validation and Evaluation of the Primers and Probes

All primers and probes included in this study were in silico analyzed for the specificity using the BLAST search tool [21]. They were also analyzed using the online IDT oligo Analyzer 3.1 tool (https://www.idtdna.com/calc/analyzer) (accessed on 21 December 2020) to check the presence of any secondary structure and primer-dimers. At this website, the tendency and stability for self- or heterodimerization can be estimated by calculating the Gibbs Free Energy (ΔG value), which is the energy required to break the secondary structure [20]. At the same time, for the in silico analysis and modification of the current assay’s probe, the IDT Custom qPCR probes online tool (https://www.idtdna.com/pages/products/qpcr-and-pcr/custom-probes) (accessed on 21 December 2020) was used.

#### 2.6.2. Melting Curve Analysis for Confirmation of Any Non-Specific Amplification

QuantiNova SYBR^®^ Green PCR Kit (QIAGEN, Hilden, Germany) was used for the identification of any non-specific amplification or primer-dimers formation within any of the tested assays. Cycling conditions for the DNA-binding dye qPCR and melting curve analysis were as follows: one cycle of pre-denaturation at 95 °C for 2 min, 40 cycles of denaturation of 95 °C for 5 s, and an annealing/extension step at 60 °C for 29 s. Melting curves were recorded after the run by stepwise temperature increase from 60 °C to 95 °C.

#### 2.6.3. Analytical Validation and Evaluation of the qPCR Assays

A. Analytical specificity: The ability of the qPCR assays to detect different isolates and serotypes of ORT (Inclusivity testing) was evaluated by testing the assays against thirty-eight ORT bacterial isolates representing ten different ORT serotypes (Table 2). On the other hand, running the assays against a panel of RNA or DNA from eighteen respiratory microorganisms that are known to inhabit or infect the avian respiratory tract (Table 2) were performed to check the lack of positive results from non-target pathogens (exclusivity testing). The used reaction mix contained a reverse transcriptase enzyme and the thermal profile contained a first step of 50 °C for 5 min to test against the listed RNA pathogens. Moreover, clinical samples from apparently normal healthy birds were included to exclude any cross-reactivity of the tested assays with any normal respiratory microflora.

B. Diagnostic specificity of the three assays was evaluated through testing of the qPCR assays against number of known positive and negative clinical samples (Table 3) submitted for ORT diagnosis. Diagnostic specificity = true negatives/(true negatives + false positives) × 100.

C. Analytical sensitivity (limit of detection), C_T_ cut-off value, and the limit of quantification (LOQ) was achieved through estimation of the lowest copy number that each assay could reliably detect to determine the presence or absence of ORT in a sample. Once the analytical sensitivity was estimated, the corresponding C_T_ value was selected as the C_T_ cut-off [37]. Additionally, limit of quantification was defined as the lowest concentration on the standard curve that maintained linearity. This was performed by:

C.1. Construction of ORT 16S rDNA positive control

A double-stranded 364 bp gBlock fragment (gBlock) for the 16S rDNA target segment, containing the forward, reverse primers, and probes sequences (of the three assays), was designed and ordered from IDT (Integrated DNA Technologies, Coralville, IA, USA).

The insert was then cloned into pCR^®^ -Blunt II TOPO^®^ (Invitrogen™) using the manufacturer’s recommendations. Briefly, gBlock insert was rehydrated in Tris-EDTA buffer (Invitrogen™) to produce a concentration of 25 ng/µL. Four µLs of the gBlock suspension was mixed with 1 µL pCR^®^-Blunt II TOPO^®^ vector and 1 µL salt solution and left at room temperature for 5 min for ligation. A three µL ligation mix was then used to transform one-shot TOP10 chemically competent cells (Invitrogen™) and grown overnight in ampicillin agar plates separately at 37 °C. The next day, a white colony was picked from the plate and grown overnight in ampicillin broth, after which the plasmid was extracted using QIAprep Spin Miniprep Kit (QIAprep^®^). The plasmid was sequenced to confirm the presence of the intended insert and subsequently converted to copy number after being quantified with the qubit fluorometric analysis double-stranded DNA high sensitivity (HS) scheme kit (Invitrogen™) using the following equation:

$$\mathrm{Number}\;\mathrm{of}\;\mathrm{copies}=\frac{X\frac{\mathrm{ng}}{µL}\times 6.0221\times 10^{23\;}\mathrm{molecules}/\mathrm{mol}}{\left(N\times \frac{660g}{\mathrm{mol}}\right)\times \;1\times 10^{9}\;\mathrm{ng}/g}$$

X = Qubit read (ng/µL).

N = length of the insert.

660 g/mol = average mass of one bp dsDNA.

The positive control was then stored at −80 °C until further use.

C.2. Generation of standard curves and estimation of the limit of detection for the three assays

Ten-fold serial dilutions of the constructed positive control DNA containing (1 × 10^10^–1 × 10^1^) copies/mL were conducted to generate the standard curve, and 5 µL was used as a template in each reaction. Average threshold cycle (C_T_) values were obtained from three independent qPCR runs; each run contained four replicates and was used to estimate the analytical sensitivity of the assays.

The average C_T_ values were plotted against log_10_ of ten-fold serial dilutions of plasmid DNA (copy number/mL), and linear equations were generated with R^2^ values.

D. Efficiency (E) was calculated by estimating the percentage of target molecules that doubled in one PCR cycle. The assays’ overall efficiency was estimated using the standard curve slope, as presented in the following equation: Efficiency = (10^(−1/slope)^ − 1) × 100

E. Coefficient of Determination (R^2^): This coefficient was used to measure the closeness of the relationship between two variables. In qPCR evaluation, it provides an indication of the consistency of serial dilutions and pipetting errors.

F. Linear dynamic range is the range of C_T_ values over which the qPCR reaction is linear. It was calculated as the range between the highest and lowest point within the standard curve for which acceptable linearity (R^2^ ≥ 0.98) and efficiency (between 90–110%) were observed.

G. Repeatability (intra-assay variation) and reproducibility (inter-assay variation): To evaluate the repeatability, each single qPCR run for each assay contained four replicates of each ten-fold serial dilution. The repeatability was then analyzed based on the standard deviation (SD) and the coefficient of variability (CV) of the C_T_ average. CV was determined by dividing the SD by the average of the obtained C_T_ values for each ten-fold serial dilution. On the other hand, to evaluate the reproducibility, each dilution of the standard curve for each assay was tested in three independent qPCR runs. All validation runs were performed on different days. The reproducibility was then analyzed based on the SD and the CV of the C_T_ average. CV was determined by dividing the SD by the average of the obtained C_T_ values.

Comparison among C_T_ values obtained from the three qPCR assays: C_T_ values generated from testing nine positive ORT clinical samples were compared among three qPCR assays (Table 3). One-way repeated ANOVA was used to assess significant differences between assays.

## 3. Results

### 3.1. Primers and Probe Design

The primers and probe of the newly developed assay were designed to target a specific segment within the 16S rRNA gene. Forward and reverse primers were designed to amplify a 131 bp segment from nt number 385,685 to 385,815 (numbering according to accession number NZ_CP006828.1).

### 3.2. In Silico Validation and Evaluation of the Primers and Probes of the Three Assays

As a first step in the in silico analysis of all primers and probes included in this study, BLAST specificity analysis was conducted. All oligonucleotides showed high specificity to ORT sequences, and there were no hits for other microorganisms within the NCBI BLAST nt database [21,38]. The query coverage and the maximum identity of the new primers and probe were all 100% only to ORT 16S rDNA sequences with expectation value (*E*-value) ≤ 0.04, suggesting potential biological relationship.

Looking at primer-dimers (self or hetero-dimer) or primer/probe dimers formation using the online IDT oligo analyzer tool, results showed multiple artifacts within the primers and probe of the currently available ORT qPCR assay. Multiple forward self-dimers and probe self-dimers were detected. Some of these secondary structures showed strong ΔG values (equal to −11.69 kcal/mol and −13.05 kcal/mol), which exceed the acceptable ΔG value of −9 kcal/mol). These primer-dimer artifacts were also observed in the modified probe assay, as the assay has exactly the same primers/probe sequences with only one difference (removal of G from the 5′ end of the probe). Another defect in the currently available assay was the presence of G base at the 5′ end of the probe, just next to the reporter.

On the other hand, no significant primer-dimers or probe-dimers were detected in the newly designed primers and probe.

### 3.3. Melting Curve Analysis for Confirmation of Any Non-Specific Amplification

**Figure 1:**
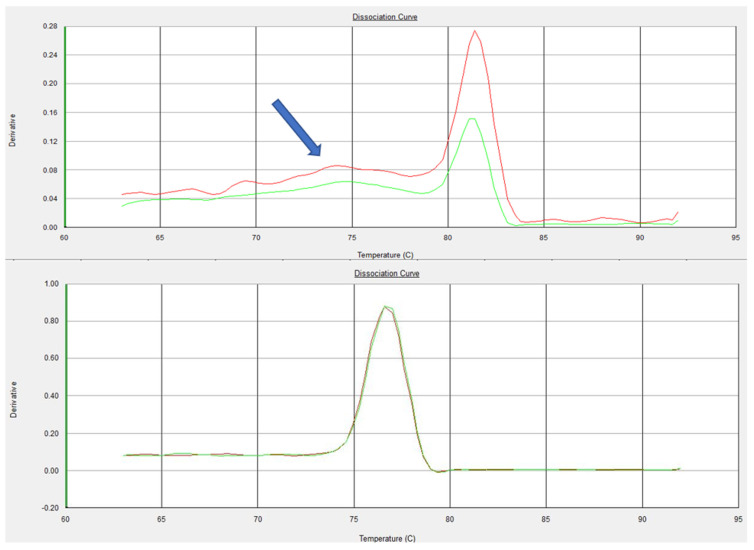
Melting curve plots displaying data collected during the melting curve analysis of tested primers. (**A**) Two peaks in the melting curve were observed during the analysis of the primer pair of the currently available assay, indicating non-specific amplification (Arrow). (**B**) Single specific peak was observed during the analysis of the newly designed primers, indicating the absence of any off-target amplification.

The formation of a secondary structure leading to amplification of non-specific amplification during the currently available assay can be observed through the presence of two melt curve peaks (Figure 1A) during the melting curve analysis. On the other hand, only a single specific peak was demonstrated during the melting curve analysis of the primer pair of the newly developed assay (Figure 1B), indicating the absence of any off-target amplification.

### 3.4. Analytical Validation and Evaluation of the qPCR Assays

#### 3.4.1. Analytical Specificity (Inclusivity and Exclusivity)

The high specificity obtained during the in silico analysis of all primers included in this study is confirmed by the high analytical specificity of the assays during the wet lab validation. All three assays could inclusively detect different ORT isolates representing ten serotypes with the complete absence of any cross-reactivity to any other tested microorganism that are likely to be found in samples submitted for ORT diagnosis (Table 2).

#### 3.4.2. Evaluation of the Assays’ Diagnostic Specificity against Clinical Samples

All three assays showed diagnostic specificity equal to 100%. All assays were able to detect only the known positive ORT clinical samples with no cross-reactivity against clinical samples from apparently normal birds (Table 3).

#### 3.4.3. Limit of Detection (LOD), C_T_ Cut-Off Value and the Limit of Quantification (LOQ)

**Figure 2:**
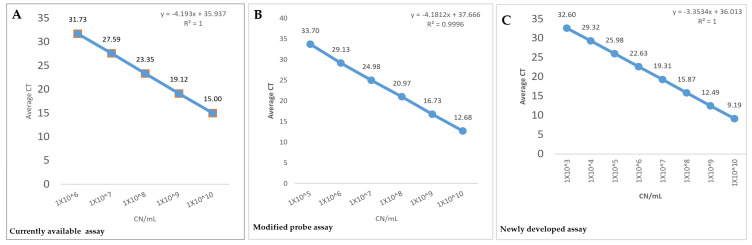
Standard curves of the three qPCR assays included in this study. (**A**): Standard curve of the current qPCR assay was generated by plotting average C_T_ values from three independent runs against log10 of 10-fold serial dilutions (10^10^–10^1^) of plasmid DNA (copy number/mL). Reaction efficiency of 73.18% was estimated using the standard curve slope. (**B**): Standard curve of the modified probe assay was generated by plotting average C_T_ values from three independent runs against log10 of 10-fold serial dilutions (10^10^–10^1^) of plasmid DNA (copy number/mL). Reaction efficiency of 73.45% was estimated using the standard curve slope. (**C**): Standard curve of the new qPCR assay was generated by plotting average C_T_ values from three independent runs against log_10_ of 10-fold serial dilutions (10^10^–10^1^) of plasmid DNA (copy number/mL). Reaction efficiency of 98.70% was estimated using the standard curve slope. Note that the newly developed assay showed improved efficiency (E = 98.70%) with approximately 1000-folds reduction in the limit of detection (from 3 × 10^6^ plasmid DNA copies/mL for the currently available assay to 1 × 10^3^ plasmid DNA copies/mL for the newly developed assay).

The newly developed ORT qPCR assay showed significant improvement in the limit of detection (approximately 1 × 10^3^ plasmid DNA Copies/mL), which was approximately 1000 folds less than the currently available assay, as shown in Table 4 and Figure 2. The C_T_ cut-off values for the currently available assay, modified probe assay, and the newly developed assay were determined to be ≤32, ≤35, and ≤33, respectively. Moreover, all of the three assays maintained linearity all the way to their LOD. As a result, the LOD and LOQ were the same for the three assays.

#### 3.4.4. Coefficient of Determination (R^2^)

Plotting average C_T_ values from three independent runs against log_10_ of 10-fold serial dilutions (10^10^–10^3^) of plasmid DNA (copy number/mL) of the three assays generated a linear equation with an R^2^ equal to (1) for both the newly developed assay and the currently available assay (Table 4). Meanwhile, the probe modified assay showed an R^2^ equal to (0.999). R^2^ > 0.98 is acceptable for well-designed qPCR assays [39], which indicates the consistency of serial dilutions.

#### 3.4.5. Efficiency (E)

Using the slope from the linear equation generated from the standard curve, the overall efficiency of the currently available assay was equal to 73.18%, despite being reported as being equal to 100% by the authors of the assay [10]. Additionally, the efficiency of the modified probe and the newly developed assay were equal to 73.45% and 98.70%, respectively (Table 4 and Figure 2). This indicates that the modification of the probe did not improve the efficiency. Additionally, only the newly developed assay had an acceptable efficiency, while the other two assays showed low efficiency below the acceptable limit (90–110%). The presence of off-target amplification artifacts of these two assays might explain the lowered efficiency and increased limit of detection.

#### 3.4.6. Linear Dynamic Range

The newly developed assay showed a wide dynamic range (from C_T_ 9.39 to C_T_ 32.97) while maintaining amplification linearity of at least eight magnitudes (Table S1–supplementary Data and Figure 2). On the other hand, the dynamic range of the other two assays could not be determined due to the lower efficiency of the assays (about 73%), which was below the acceptable limit of 90%.

#### 3.4.7. Repeatability

The intra-assay coefficient of variability (%CV) for the C_T_ values determined for the newly developed assay ranged from (0.40% to 1.89%), with an average = 0.98%. On the other hand, the %CV for the currently available assay ranged from (0.1% to 1.27%) with an average = 0.64%, while the probe modified assay showed %CV ranged from (0.39% to 0.93%) with an average = 0.77% (Table S1–supplementary Data). These values demonstrate good repeatability of the three assays, where %CV less than 10% is acceptable for intra-assay variability [40].

#### 3.4.8. Reproducibility

The inter-assay %CV for the C_T_-values determined for the newly developed qPCR ranged from (0.65% to 3.11%) with an average = 1.53%. On the other hand, %CV for the currently available assay ranged from (3.13% to 3.66%) with an average = 3.33%, while the inter-assay %CV for the modified probe qPCR ranged from (2.23% to 4.12%) with an average = 2.9% (Table S1—supplementary Data). These values reveal the acceptable reproducibility of the three assays, where %CV less than 15% is acceptable for inter-assay variability [40].

### 3.5. Comparison among C_T_ Values Obtained from Testing Positive Clinical Samples Using the Three qPCR Assays

The improved LOD and E of the new assay as a reflection of the improved design of primers and probe led to statistically significant differences in C_T_ values generated from testing known positive ORT clinical samples. The newly designed assay showed values ranged from 4.45 to 9 lower C_T_ when testing the same sample, with an average C_T_ difference = 7.12 in comparison with the currently available assay. Additionally, the modified probe assay showed earlier C_T_ values ranged from 2.52 to 6.14, with an average C_T_ difference = 3.50 than the currently available ORT qPCR assay (Table 3). Similar significant C_T_ value differences were observed form isolates and cloned gBlock insert.

## 4. Discussion

Real-time PCR has multiple advantages over the traditional culture-dependent methods, including improved sensitivity and specificity and less turnaround time [41]. In addition to that, working with a fastidious microorganism such as ORT that is easily overgrown by other bacteria adds great value to the PCR as an essential diagnostic tool. Two PCR assays for ORT detection have been published previously. One is conventional and the second one is TaqMan qPCR [6,10]. However, during the validation of the currently available qPCR assay [10], multiple issues were observed that led to sub-optimal performance (e.g., low efficiency and low analytical sensitivity). In this manuscript, we describe both probe modification of the current assay along with the design of a new qPCR assay in an attempt to improve the available molecular diagnostics of ORT. Subsequently, a comparison between the performances of the three assays was then carried out.

During the in silico analysis of the primers and probe of the current assay [10], the primers showed high specificity to ORT sequences. However, a number of artifacts were observed during the oligo analysis. For instance, the presence of G base was observed at the 5′ end of the probe adjacent to the reporter dye. Compared with the other three nucleobases, G has the lowest oxidation potential, which means that G can be oxidized more easily [42] and will act as a super quencher to the fluorophore [42,43]. Moreover, its location at the 5′ end will decrease the hydrolysis of the probe [18]. Multiple reports have demonstrated that G at the 5′ of the probe would affect the performance of any qPCR assay [18,39].

A probe modification was carried out by removing the G from the 5′ end of the probe of the currently available assay. An evaluation of the effect of probe modification on the assay performance was then implemented. Although this modification improved the performance over the currently available assay (analytical sensitivity improved from 3 × 10^6^ plasmid DNA copies/mL to 3 × 10^5^ plasmid DNA copies/mL), the modified probe assay’s performance was still substandard (showed lower efficiency of 73.45% and LOD equals to approximately 1 × 10^5^ plasmid DNA copies/mL). This was an indication that the issues with this assay’s performance were bigger than the mere presence of a G at the 5′ end of the probe.

Multiple primer design programs use ΔG to measure the spontaneity of formation of the most stable dimer [44,45,46,47,48]. Further in silico analysis of the primers and probe of the currently available ORT qPCR revealed the formation of primer-dimers with large ΔG negative values. These stable primer-dimers can competitively reduce binding to target DNA and subsequently reduce the amplification efficiency, and subsequently, the sensitivity of the assay [49].

Despite the fact that in silico tools provide valuable feedback, we wanted to confirm these results for primer-dimer formation through running melting curve analysis using DNA intercalating dye as recommended [50,51]. After PCR, a gradual increase in temperature was applied, leading to the melting of the annealed products. The decrease in fluorescence was recorded as the strands dissociated [52]. Every sequence has its signature melting temperature, which allows the confirmation of any off-target amplification. Melting curve analysis with two peaks confirmed the presence of off-target amplification for the current assay (Figure 1A), and this is why we developed a new assay.

Therefore, we adopted the general concepts for qPCR primers and probe design [53], and a new set of primers and probe targeting the same gene (16S rRNA) were designed. BLAST analysis of the newly designed oligos showed high specificity only to ORT sequences with small *E*-value. In performing the melting curve analysis for the newly designed assay, a single specific peak confirmed the absence of any off-target amplification (Figure 1B).

The absence of any primer-dimers for the newly designed assay during the in silico analysis and melting curve analysis led to significant improvement in the LOD and efficiency. On the other hand, the other two assays showed off-target amplification artifacts formed by primer–primer binding or primer–probe binding resulted in reduced amplification efficiency and suboptimal product yields for the other two assays [49].

PCR efficiency is an important performance characteristic, as stated in the Minimum Information for Publication of Quantitative Real-time PCR Experiments (MIQE) guidelines [50]. The efficiency of the currently available assay [10] was reported to be 100%. During the validation of this assay, we used different reaction mix, and the amplification conditions were slightly changed from the conditions of the current assay (95 °C for 15 min, 42 cycles of 94 °C for 60 S, and 60 °C for 60 S) [10]. However, the annealing-extension step was the same “60 °C for 60 S”. These slight changes in the amplification conditions were not expected to have a noticeable impact on the performance of the assay. Additionally, the probe described in the initial manuscript for the currently available assay had a FAM-TAMRA labelled probe. In this manuscript, we labeled the probe of the currently available assay as well as the two other assay using FAM/internal quencher ZEN/3IABkFQ. By switching to the new quencher system, we expected an improvement in the assay performance, lower overall background, and lower CT values. This was due to the difference in the quenching mechanism. TAMRA is a fluorescent quencher that absorbs the light emitted by FAM and releases it as light. On the other hand, both ZEN and Iowa Black-FQ are non-fluorescent quenchers (NFQs). These quenchers will absorb the light released by FAM and release it as heat and the overall background signal will be lower. Because the background signal is lower, the FAM signal will cross the critical threshold sooner, giving lower C_T_ values. However, the observed efficiency and subsequently the sensitivity of the current assay were significantly lower (≈73%) than the efficiency reported in the initial manuscript [10]. The decreased efficiency and sensitivity of the currently available assay reported in this current manuscript could be corroborated and explained by the detection of stable primer-dimers during the in silico and melting curve analysis. The lowered efficiency of the currently available and modified probe assay (about 73%) below the acceptable limit (90%) led to the inability to calculate the linear dynamic range of the two assays. On the other hand, the newly designed assay showed a broad linear dynamic range of at least eight orders of magnitude (from 3 × 10^3^ plasmid DNA copies/mL to 3 × 10^10^ plasmid DNA copies/mL) with acceptable linearity (R^2^ =1) and efficiency (E = 98.70%).

Describing key quality control parameters such as R^2^ is essential for the correct interpretation of qPCR results [50]. All of the three assays showed R^2^ ≥ 0.998. An R^2^ value > 0.980 provides confidence in correlating C_T_ values and target copy number [53]. Moreover, to be implemented as a reliable diagnostic test, the qPCR assay should be repeatable and reproducible. All three assays showed a good level of repeatability and reproducibility even at the highest dilutions. However, better primers and probe design and better efficiency of the newly developed assay are reflected as lower C_T_ values and improved analytical sensitivity, therefore lower LOD (Table 3 and Table 4, Figure 2). For instance, the newly developed assay showed acceptable efficiency (E = 98.70%) with approximately 1000-folds reduction in the limit of detection (from 3 × 10^6^ plasmid DNA copies/mL for the currently available assay to 1 × 10^3^ plasmid DNA copies/mL for the newly developed assay). Additionally, the improved efficiency of the new assay led to lower C_T_ values (average = 7.12) than the current assay when clinical samples were tested. However, despite validating the assay against nine known positive clinical samples, using it on a larger number of clinical samples will be the ultimate confirmation of its suitability for clinical applications.

## 5. Conclusions

The newly developed assay is an improvement upon the currently available assay and will improve our diagnostic capabilities of detecting ORT from clinical samples. This in turn will increase our confidence in achieving accurate ORT diagnosis, which is essential in understanding this disease’s epidemiology and developing effective prevention, control, and eradication methods.

## Figures and Tables

**Table 1:** Oligonucleotides characteristics of the three ORT TaqMan qPCR assays included in this study.

Oligo	Sequence (5′ to 3′)	Length (bp)	Nt Position ^a^	Amplified Segment Length	Reference
Forward Primer	GAG AAT TAA TTT TCG GAT TAA G	22	385,848–385,869	119 bp	Currently available assay [10]
Reverse Primer	CAA TCA AAA TCT TAT GGA GT	20	385,751–385,770		
Probe	FAM **G**TA ACG CGT/ZEN/ATG CAA CTT GC 3IABkFQ ^b^	20	385,809–385,828		
Forward Primer	GAGAATTAATTTTCGGATTAAG	22	385,848–385,869	119 bp	Modified probe assay (This study)
Reverse Primer	CAATCAAAATCTTATGGAGT	20	385,751–385,770		
Probe	FAM TAA CGC GTA/ZEN/TGC AAC TTG C 3IABkFQ	19	385,809–385,827		
Forward Primer	CTA CCA ACT AAC TAA TCT GAC GCA	24	385,685–385,708	131 bp	Newly developed assay (This study)
Reverse Primer	AAC TTG CCC TTA TCA GGA GGA T	22	385,794–385,815		
Probe	FAM CGG GGA AAC/ZEN/TCG GAT TAA TAC TCC ATA AG 3IABkFQ	29	385,761–385,789		

**^a^** Nucleotide position according to GenBank accession number (NZ_CP006828.1). Bold Red **G** is the removed base in the modified probe. **^b^** The original published probe was “FAM-TAMRA” labelled.

**Table 2:** ORT and other bacterial and viral isolates used to evaluate the analytical specificity of the assays in this study.

Sample No.	Organism	Information * (Age, Spp., and Year)	Sample Type *	Serotype	Currently Available ORT qPCR [10]	Probe Modified qPCR	Newly Developed qPCR	Growth Conditions According to
1	ORT	43 days-Chicken-2020	Isolate	-	+	+	+	[22]
2	ORT	28 days-Chicken-2020	Isolate	-	+	+	+	[22]
3	ORT	23 days-Chicken-2019	Isolate	-	+	+	+	[22]
4	ORT	26 days-Chicken-2020	Isolate	-	+	+	+	[22]
5	ORT	39 days-Chicken-2019	Isolate	-	+	+	+	[22]
6	ORT	36 days-Chicken-2019	Isolate	-	+	+	+	[22]
7	ORT	-	Isolate	-	+	+	+	[22]
8	ORT	- -1999	Isolate	-	+	+	+	[22]
9	ORT	- -Turkey-2009	Isolate	N	+	+	+	[22]
10	ORT	- -Turkey-2008	Isolate	H	+	+	+	[22]
11	ORT	- -Turkey-2009	Isolate	N	+	+	+	[22]
12	ORT	- -Turkey-2009	Isolate	N	+	+	+	[22]
13	ORT	- -Turkey-2008	Isolate	H	+	+	+	[22]
14	ORT	- -Turkey-2009	Isolate	H	+	+	+	[22]
15	ORT	- -Turkey-2009	Isolate	H	+	+	+	[22]
16	ORT	- -Turkey-2008	Isolate	-	+	+	+	[22]
17	ORT	- -Turkey-2009	Isolate	H	+	+	+	[22]
18	ORT	- -Turkey-2009	Isolate	-	+	+	+	[22]
19	ORT	- -Turkey-2008	Isolate	H	+	+	+	[22]
20	ORT	- -Turkey-2009	Isolate	H	+	+	+	[22]
21	ORT	- -Turkey-2009	Isolate	H	+	+	+	[22]
22	ORT	- -Turkey-	Isolate	H	+	+	+	[22]
23	ORT	- -Turkey- -	Isolate	H	+	+	+	[22]
24	ORT	- -1996	Isolate	-	+	+	+	[22]
25	ORT	Chicken-2019	Isolate	F	+	+	+	[22]
26	ORT	Chicken-2019	Isolate	F	+	+	+	[22]
27	ORT	Chicken-2019	Isolate	N	+	+	+	[22]
28	ORT	Chicken-2019	Isolate	J	+	+	+	[22]
29	ORT	Chicken-2014	Isolate	A	+	+	+	[22]
30	ORT	Chicken-2014	Isolate	A	+	+	+	[22]
31	ORT	Chicken-2014	Isolate	C	+	+	+	[22]
32	ORT	Chicken-2014	Isolate	C	+	+	+	[22]
33	ORT	Chicken	Isolate	D	+	+	+	[22]
34	ORT	Chicken	Isolate	L	+	+	+	[22]
35	ORT	Chicken	Isolate	G	+	+	+	[22]
36	ORT	Chicken	Isolate	G	+	+	+	[22]
37	ORT	Chicken	Isolate	J	+	+	+	[22]
38	ORT	Chicken	Isolate	E	+	+	+	[22]
39	*Mycoplasma gallisepticum*	-	Isolate	-	–	–	–	[23]
40	*Mycoplasma iowae*	-	Isolate	-	–	–	–	[24]
41	*Mycoplasma synoviae*	-	Isolate	-	–	–	–	[23]
42	*Bordetella hinzii*	-	Isolate	-	–	–	–	[25]
43	*Pasteurella multocida*	-	Isolate	-	–	–	–	[26]
44	*Pasteurella multocida*	-	Isolate	-	–	–	–	[26]
45	*Escherichia coli*	-	Isolate	-	–	–	–	[27]
46	*Gallibacterium anatis*	-	Isolate	-	–	–	–	[28]
47	*Erysipelothrix rhusiopathiae*	-	Isolate	-	–	–	–	[29]
48	*Staphylococcus aureus*	-	Isolate	-	–	–	–	[30]
49	*Avibacterium paragallinarum*	-	Isolate	-	–	–	–	[31]
50	*Bordetella avium*	-	Isolate	-	–	–	–	[32]
51	Avian Avulavirus 1 (Newcastle Disease)	-	Isolate	-	–	–	–	[33]
52	Avian Reovirus	-	Isolate	-	–	–	–	[34]
53	Infectious Bronchitis Virus	-	Isolate	-	–	–	–	[35]
54	Infectious Bronchitis Virus	-	Isolate	-	–	–	–	[35]
55	Infectious Bronchitis Virus	-	Isolate	-	–	–	–	[35]
56	Infectious Laryngotracheitis Virus	-	Isolate	-	–	–	–	[36]

ORT Isolates were obtained from Dr. Kakambi Nagaraja, University of Minnesota. Bacterial and viral isolates other than ORT were obtained from the Bacteriology unit of Veterinary Diagnostic Laboratory, Iowa State University. * Age of animal at sampling–poultry species–year of sample collection.

**Table 3:** Known positive and negative ORT clinical samples included in this study and comparison among C_T_ values obtained from testing positive clinical samples using the three qPCR assays.

Sample No.	Information ^a^ (Age, host, and year)	Sample Type	Currently Available ORT qPCR (C_T_ value)	Probe Modified qPCR (C_T_ value)	Newly Developed qPCR (C_T_ value)
1	64 days-Turkey-2019	Oropharyngeal swab ^b^	19.47	16.95	15.02
2	63 days-Turkey-2019	Lung homogenate ^b^	27.66	24.45	19.42
3	63 days-Turkey-2019	Oropharyngeal swab ^b^	20.75	17.71	14.51
4	42 days-Turkey-2019	Tracheal homogenate ^b^	22.38	19.26	15.38
5	14 days-Turkey-2019	Tracheal swabs ^b^	27.67	21.53	19.22
6	14 days-Turkey-2019	Tracheal swabs ^b^	23.48	19.44	16.05
7	48 days-Turkey-2019	Tracheal homogenate ^b^	20.77	17.29	14.26
8	48 days-Turkey-2019	Tracheal swabs ^b^	30.80	28.02	21.80
9	392 days-Chicken-2020	Tracheal swabs ^b^	22.18	19.02	15.38
10	4.5 years-Chicken-2019	Lung homogenate ^c^	–	–	–
11	4.5 years-Chicken-2019	Tracheal homogenate ^c^	−	–	–
12	7 days-Turkey-2019	Tracheal homogenate ^c^	–	–	–
13	7 days-Turkey-2019	Lung homogenate ^c^	–	–	–
14	51 days-Turkey-2019	Lung homogenate ^c^	–	–	–
15	67 days-Turkey-2019	Lung homogenate ^c^	–	–	–
16	10 days-Turkey-2019	Tracheal homogenate ^c^	–	–	–
17	10 days-Turkey-2019	Tracheal homogenate ^c^	–	–	–
18	252 days-Chicken-2019	Lung homogenate ^c^	–	–	–
19	266 days-Chicken-2019	Lung homogenate ^c^	–	–	–
20	266 days-Chicken-2019	Tracheal homogenate ^c^	–	–	–
21	595 days-Chicken-2019	Tracheal homogenate ^c^	–	–	–
22	595 days-Chicken-2019	Lung homogenate ^c^	–	–	–
23	2 days-Turkey-2019	Lung homogenate ^c^	–	–	–
24	2 days-Turkey-2019	Tracheal homogenate ^c^	–	–	–
25	21 days-Turkey-2019	Lung homogenate ^c^	–	–	–
26	21 days-Turkey-2019	Tracheal homogenate ^c^	–	–	–

^a^ Age of animal at sampling–poultry species–year of sample collection. ^b^ Known positive ORT clinical sample. ^c^ Known negative ORT clinical sample. Positive and negative clinical samples were obtained from clinical cases submitted to the Veterinary Diagnostic Laboratory, Iowa State University. All three assays showed 100% diagnostic specificity. However, the newly developed assay showed consistently lower C_T_ values in comparison with the other two assays.

**Table 4:** Limits of detection and standard curve results of the three qPCRs.

qPCR Assay	Target Gene	Amplicon Size	Limit of Detection	Linear Equation	R^2^	Efficiency
Current assay	16S rRNA	119 bp	1 × 10^6^ copies/mL	y = −4.193x + 35.937	R² = 1	E = 73.18 %
Modified probe assay		119 bp	1 × 10^5^ copies/mL	y = −4.1812x + 37.666	R² = 0.999	E = 73.45%
Newly developed assay		131 bp	1 × 10^3^ copy/mL	y = −3.3534x + 36.013	R² = 1	E = 98.70%

## Supplementary Materials

The following are available online at https://www.mdpi.com/2076-2607/10/2/341/s1, Table S1: Intra- and inter-assay variations in different concentrations of positive control DNA of the three ORT qPCRs; Table S2: Average CT values generated from three independent qPCR runs using the currently available assay. Average CT values were then used for the calculation of R2 and generation of standard curve equation; Table S3: Average C_T_ values generated from three independent qPCR runs using the modified probe assay. Average C_T_ values were then used for the calculation of R^2^ and generation of standard curve equation; Table S4: Average C_T_ values generated from three independent qPCR runs using the newly developed assay. Average C_T_ values were then used for the calculation of R^2^ and generation of standard curve equation.

## References

[1] Glisson J.R. (1998). Bacterial respiratory disease of poultry. Poult. Sci..

[2] Van Empel P., Hafez H. (1999). Ornithobacterium rhinotracheale: A review. Avian Pathol..

[3] Churria C.D.G., Machuca M.A., Petruccelli M.A. (2012). Ornithobacterium rhinotracheale infection in poultry: An updated review. Int. J. Mol. Zool..

[4] Clark S. Current health and industry issues facing the turkey industry. Proceedings of the Annual meeting of the United States Animal Health Association 2018.

[5] Barbosa E.V., Cardoso C.V., Silva R.D.C.F., Cerqueira A.D.M.F., Liberal M.H.T., Castro H.C. (2020). Ornithobacterium rhinotracheale: An Update Review about An Emerging Poultry Pathogen. Vet. Sci..

[6] Empel V. (1998). Ornithobacterium Rhinotracheale.

[7] Hafez H.M. (2002). Diagnosis of Ornithobacterium rhinotracheale. Int. J. Poult. Sci..

[8] Van Veen J.N.L., Mekkes D., Vrijenhoek M., van Empel P. (2005). Diagnosis and incidence of Ornithobacterium rhinotracheale infections in commercial broiler chickens at slaughter. Vet. Rec..

[9] Ha H.J., Christensen N., Humphrey S., Haydon T., Bernardi G., Rawdon T. (2016). The first detection of Ornithobacterium rhinotracheale in New Zealand. Avian Dis..

[10] Abdelwhab E., Lüschow D., Hafez H. (2013). Development of real-time polymerase chain reaction assay for detection of Ornithobacterium rhinotracheale in poultry. Avian Dis..

[11] Canal C.W., Leao J.A., Rocha S.L.S., Macagnan M., Lima-Rosa C.A.V., Oliveira S.D., Back A. (2005). Isolation and characterization of Ornithobacterium rhinotracheale from chickens in Brazil. Res. Vet. Sci..

[12] Chansiripornchai N., Wanasawaeng W., Sasipreeyajan J. (2007). Seroprevalence and identification of Ornithobacterium rhinotracheale from broiler and broiler breeder flocks in Thailand. Avian Dis..

[13] Koga Y., Zavaleta A.I. (2005). Intraspecies genetic variability of Ornithobacterium rhinotracheale in commercial birds in Peru. Avian Dis..

[14] Tsai H.-J., Huang C.-W. (2006). Phenotypic and molecular characterization of isolates of Ornithobacterium rhinotracheale from chickens and pigeons in Taiwan. Avian Dis..

[15] Arif M., Aguilar-Moreno G.S., Wayadande A., Fletcher J., Ochoa-Corona F.M. (2014). Primer modification improves rapid and sensitive in vitro and field-deployable assays for detection of high plains virus variants. Appl. Environ. Microbiol..

[16] Li D., Zhang J., Li J. (2020). Primer design for quantitative real-time PCR for the emerging Coronavirus SARS-CoV-2. Theranostics.

[17] Stenzel T., Pestka D., Tykałowski B., Śmiałek M., Koncicki A., Bancerz-Kisiel A. (2017). Detection of Bordetella avium by TaqMan real-time PCR in tracheal swabs from wildlife birds. Pol. J. Veter-Sci..

[18] Lunge V., Miller B., Livak K., Batt C. (2002). Factors affecting the performance of 5′ nuclease PCR assays for Listeria monocytogenes detection. J. Microbiol. Methods.

[19] Crockett A.O., Wittwer C.T. (2001). Fluorescein-Labeled Oligonucleotides for Real-Time PCR: Using the Inherent Quenching of Deoxyguanosine Nucleotides. Anal. Biochem..

[20] Johnston A.D., Lu J., Ru K., Korbie D., Trau M. (2019). PrimerROC: Accurate condition-independent dimer prediction using ROC analysis. Sci. Rep..

[21] Altschul S.F., Gish W., Miller W., Myers E.W., Lipman D.J. (1990). Basic local alignment search tool. J. Mol. Biol..

[22] Smith E.A., Miller E.A., Weber B.P., Aguayo J.M., Figueroa C.F., Huisinga J., Nezworski J., Kromm M., Wileman B., Johnson T.J. (2020). Genomic landscape of Ornithobacterium rhinotracheale in commercial turkey production in the United States. Appl. Environ. Microbiol..

[23] Ball C., Felice V., Ding Y., Forrester A., Catelli E., Ganapathy K. (2020). Influences of swab types and storage temperatures on isolation and molecular detection of Mycoplasma gallisepticum and Mycoplasma synoviae. Avian Pathol..

[24] Singh P., Yavari C.A., Newman J.A., Bradbury J.M. (1997). Identification of Mycoplasma Iowae by Colony Immunoblotting Utilizing Monoclonal Antibodies. J. Veter-Diagn. Investig..

[25] Register K.B., Kunkleb R.A. (2009). Strain-Specific Virulence of Bordetella hinzii in Poultry. Avian Dis..

[26] Mbuthia P.G., Njagi L.W., Nyaga P.N., Bebora L.C., Minga U., Kamundia J., Olsen J.E. (2008). Pasteurella multocida in scavenging family chickens and ducks: Carrier status, age susceptibility and transmission between species. Avian Pathol..

[27] Sarba E.J., Kelbesa K.A., Bayu M.D., Gebremedhin E.Z., Borena B.M., Teshale A. (2019). Identification and antimicrobial susceptibility profile of Escherichia coli isolated from backyard chicken in and around ambo, Central Ethiopia. BMC Veter-Res..

[28] Nassik S., Tallouzt S., Karbach N., Touzani C., Bidoudan Y., Aamarine N., Hess C. (2019). First Report of Isolation of Gallibacterium anatis from Layer Chickens in Morocco with Decrease in Laying Performance. Avian Dis..

[29] Eriksson H., Bagge E., Båverud V., Fellström C., Jansson D.S. (2014). Erysipelothrix rhusiopathiae contamination in the poultry house environment during erysipelas outbreaks in organic laying hen flocks. Avian Pathol..

[30] Krupa P., Bystroń J., Bania J., Podkowik M., Empel J., Mroczkowska A. (2014). Genotypes and oxacillin resistance of Staphylococcus aureus from chicken and chicken meat in Poland. Poult. Sci..

[31] Christensen H.B.P., Bisgaard M., Louise D.-Z., Williams S.M., Jackwood M.W., Lee M.D., Lupiani B., Reed W.M., Spackman E., Woolcock P.R. (2016). Pasteurella, Avibacterium, Gallibacterium Species. A Laboratory Manual for the Isolation, Identification, and Characterization of Avian Pathogens.

[32] Hashish A., Sinha A., Mekky A., Sato Y., Macedo N.R., El-Gazzar M. (2021). Development and Validation of Two Diagnostic Real-Time PCR (TaqMan) Assays for the Detection of Bordetella avium from Clinical Samples and Comparison to the Currently Available Real-Time TaqMan PCR Assay. Microorganisms.

[33] Msoffe P.L.M., Chiwanga G.H., Cardona C.J., Miller P.J., Suarez D.L. (2019). Isolation and Characterization of Newcastle Disease Virus from Live Bird Markets in Tanzania. Avian Dis..

[34] Jones R. (2000). Avian reovirus infections. J. Rev. Sci. Et Tech.-Off. Int. Des Epizoot..

[35] Gelb J., Jackwood M., Swayne D.E., Glisson J.R., Jackwood M.W., Pearson J.E., Reed W.M. (1998). Infectious bronchitis. A Laboratory Manual for the Isolation Identification of Avian Pathogens.

[36] Dormitorio T.V., Giambrone J.J., Macklin K. (2013). Detection and Isolation of Infectious Laryngotracheitis Virus on a Broiler Farm After a Disease Outbreak. Avian Dis..

[37] Caraguel C.G.B., Stryhn H., Gagné N., Dohoo I.R., Hammell K.L. (2011). Selection of a cutoff value for real-time polymerase chain reaction results to fit a diagnostic purpose: Analytical and epidemiologic approaches. J. Vet. Diagn. Invest..

[38] National Center for Biotechnology Information The Nucleotide Database. https://blast.ncbi.nlm.nih.gov/Blast.cgi?PROGRAM=blastn&PAGE_TYPE=BlastSearch&LINK_LOC=blasthome.2021[cited.

[39] Behlke M.A., Huang L., Bogh L., Rose S., Devor E.J. (2005). Fluorescence quenching by proximal G-bases. Integr. DNA Technol..

[40] Calculating Inter- and Intra-Assay Coefficients of Variability. https://salimetrics.com/calculating-inter-and-intra-assay-coefficients-of-variability.

[41] Balakrishnan B., Luckey D., Marietta E., Karau M., Patel R., Murray J., Taneja V. (2017). Development of a real-time PCR method for quantification of Prevotella histicola from the gut. Anaerobe.

[42] Fan X., Lin F., Zhang Y., Zhao J., Li H., Yao S. (2012). A simple adenosine fluorescent aptasensor based on the quenching ability of guanine. New J. Chem..

[43] Torimura M., Kurata S., Yamada K., Yokomaku T., Kamagata Y., Kanagawa T., Kurane R. (2001). Fluorescence-Quenching Phenomenon by Photoinduced Electron Transfer between a Fluorescent Dye and a Nucleotide Base. Anal. Sci..

[44] Marshall O.J. (2004). PerlPrimer: Cross-platform, graphical primer design for standard, bisulphite and real-time PCR. Bioinformatics.

[45] Owczarzy R., Tataurov A.V., Wu Y., Manthey J.A., McQuisten K.A., Almabrazi H.G., Pedersen K.F., Lin Y., Garretson J., McEntaggart N.O. (2008). IDT SciTools: A suite for analysis and design of nucleic acid oligomers. Nucleic Acids Res..

[46] Qu W., Zhou Y., Zhang Y., Lu Y., Wang X., Zhao D., Yang Y., Zhang C. (2012). MFEprimer-2.0: A fast thermodynamics-based program for checking PCR primer specificity. Nucleic Acids Res..

[47] Rychlik W. (2007). OLIGO 7 primer analysis software. PCR Primer Des..

[48] Untergasser A. (2012). Primer3—New capabilities and interfaces. Nucleic Acids Res..

[49] Rychlik W. (1993). Selection of Primers for Polymerase Chain Reaction. PCR Protocols.

[50] Bustin S.A. (2009). The MIQE Guidelines: Minimum Information for Publication of Quantitative Real-Time PCR Experi-ments. Clin. Chem..

[51] Raymaekers M., Smets R., Maes B., Cartuyvels R. (2009). Checklist for optimization and validation of real-time PCR assays. J. Clin. Lab. Anal..

[52] Pryor R.J., Wittwer C.T., Lo Y.M.D., Chiu R.W.K., Chan K.C.A. (2006). Real-Time Polymerase Chain Reaction and Melting Curve Analysis. Clinical Applications of PCR.

[53] Bustin S., Huggett J. (2017). qPCR primer design revisited. Biomol. Detect. Quantif..

