# Correction: Hashish et al. Development and Validation of a New TaqMan Real-Time PCR for the Detection of *Ornithobacterium rhinotracheale*. *Microorganisms* 2022, *10*, 341

**DOI:** 10.3390/microorganisms10050917

**Published:** 2022-04-27

**Authors:** Amro Hashish, Avanti Sinha, Yuko Sato, Nubia R. Macedo, Mohamed El-Gazzar

**Affiliations:** 1 Department of Veterinary Diagnostic and Production Animal Medicine, College of Veterinary Medicine, Iowa State University, Ames, IA 50011, USA; hashish@iastate.edu (A.H.); asinhac@gmail.com (A.S.); ysato@iastate.edu (Y.S.); nubia@iastate.edu (N.R.M.); 2 Agriculture Research Center, National Laboratory for Veterinary Quality Control on Poultry Production, Animal Health Research Institute, Giza 12618, Egypt

The authors would like to make the following corrections to this paper [1]:

There is a mistake in the sequence of the reverse primer of the newly developed assay provided in Table 1. The sequence of the reverse primer reported in the original version of the manuscript was: 5′ AAC TTG CCC TTA TCA GGA T 3′. The correct sequence of the reverse primer for the newly developed assay is: 5′ AAC TTG CCC TTA TCA GGA GGA T 3′.
