# Supplementary material for: Development and Validation of a New TaqMan Real-Time PCR for the Detection of Ornithobacterium rhinotracheale
Source: Microorganisms. 2022 Feb 1;10(2):341. doi: 10.3390/microorganisms10020341 (PMC8875355; doi:10.3390/microorganisms10020341)
Supplement: Supplementary file 1 [file microorganisms-10-00341-s001.zip › microorganisms-1522184-supplementary.pdf]

microorganisms
MDPI

Supplementary materials

Table S1. Intra- and inter-assay variations in different concentrations of positive control DNA of the three ORT qPCRs.

Copy number/mL	Repeatability			Reproducibility			Linear dynamic Range
	Mean C_T	SD	CV%	Mean C_T	SD	CV%	
**Currently available qPCR**							
1.0E+02	-	-	-	-	-	-	Couldn't be determined due to the lower efficiency of the assay (E = 73.18%) below the acceptable range (90-110%).
1.0E+03	-	-	-	-	-	-	
1.0E+04	-	-	-	-	-	-	
1.0E+05	-	-	-	-	-	-	
1.0E+06	31.99	0.29	0.92	31.73	0.99	3.13	
1.0E+07	27.83	0.09	0.33	27.59	1.01	3.66	
1.0E+08	23.58	0.02	0.10	23.35	0.73	3.14	
1.0E+09	19.34	0.25	1.27	19.12	0.63	3.30	
1.0E+10	15.21	0.09	0.59	15.00	0.51	3.43	
Modified probe qPCR							
1.0E+02	-	-	-	-	-	-	Couldn't be determined due to the lower efficiency of the assay (E = 73.45%) below the acceptable range (90-110%).
1.0E+03	-	-	-	-	-	-	
1.0E+04	-	-	-	-	-	-	
1.0E+05	34.81	0.30	0.86	33.70	1.39	4.12	
1.0E+06	29.62	0.23	0.76	29.13	0.86	2.95	
1.0E+07	25.43	0.24	0.93	24.98	0.70	2.79	
1.0E+08	21.34	0.08	0.39	20.97	0.63	2.99	
1.0E+09	16.90	0.15	0.87	16.73	0.39	2.34	
1.0E+10	12.72	0.10	0.82	12.68	0.28	2.23	
Newly developed qPCR							
1.0E+02	-	-	-	-	-	-	Wide linear dynamic range while maintaining amplification linearity of at least eight magnitudes (from C_T 12.49 to C_T 32.60)
1.0E+03	32.97	0.17	0.51	32.60	0.34	1.04	
1.0E+04	29.54	0.12	0.40	29.32	0.19	0.65	
1.0E+05	26.25	0.22	0.83	25.98	0.24	0.91	
1.0E+06	22.93	0.22	0.97	22.63	0.26	1.15	
1.0E+07	19.56	0.26	1.32	19.31	0.22	1.13	
1.0E+08	16.03	0.17	1.03	15.87	0.14	0.88	
1.0E+09	12.75	0.24	1.89	12.49	0.24	1.89	
1.0E+10	9.39	0.08	0.88	9.19	0.29	3.11	

The table revealed that all of the three assays showed a good level of repeatability (CV <10%) and reproducibility (CV <15%). However, the new assay showed a broad linear dynamic range of at least eight orders of magnitude, while the dynamic range of the other two assays could not be calculated due to lowered efficiency.

Table S2. Average C_T values generated from three independent qPCR runs using the currently available assay. Average C_T values were then used for the calculation of R^2 and generation of standard curve equation.

	Run1	Run2	Run3	
CN/mL	Mean C_T Value	Mean C_T Value	Mean C_T Value	Average C_T
1×10^5				
1×10^6	32.57	30.64	31.99	31.73
1×10^7	28.46	26.48	27.83	27.59
1×10^8	23.94	22.53	23.58	23.35
1X10^9	19.62	18.41	19.34	19.12
1X10^10	15.37	14.41	15.21	15.00

Table S3. Average C_T values generated from three independent qPCR runs using the modified probe assay. Average C_T values were then used for the calculation of R^2 and generation of standard curve equation.

	Run1	Run2		Run3
CN/mL	Mean C_T Value	Mean C_T Value	Mean C_T Value	Average C_T
1×10^5	34.15	32.15	34.81	33.70
1×10^6	29.63	28.14	29.62	29.13
1×10^7	25.33	24.18	25.43	24.98
1×10^8	21.33	20.25	21.34	20.97
1×10^9	17.00	16.28	16.90	16.73
1×10^10	12.94	12.37	12.72	12.68

Table S4. Average C_T values generated from three independent qPCR runs using the newly developed assay. Average C_T values were then used for the calculation of R^2 and generation of standard curve equation.

	Run1	Run2	Run3	
CN/mL	Mean C_T Value	Mean C_T Value	Mean C_T Value	Average C_T
1.0E+03	32.52	32.97	32.30	32.60
1.0E+04	29.24	29.54	29.18	29.32
1.0E+05	25.87	26.25	25.82	25.98
1.0E+06	22.48	22.93	22.47	22.63
1.0E+07	19.18	19.56	19.19	19.31
1.0E+08	15.81	16.03	15.77	15.87
1.0E+09	12.29	12.75	12.41	12.49
1.0E+10	9.19	9.39	8.99	9.19
